# Home screening of taste and oral trigeminal function: a feasibility study

**DOI:** 10.1007/s00405-024-08654-5

**Published:** 2024-04-17

**Authors:** Tomer Green, Mariano Mastinu, Anne Wolf, Anna Oleszkiewicz, Anna Aronis, Thomas Hummel, M. Yanina Pepino, Masha Y. Niv

**Affiliations:** 1 Institute of Biochemistry, Food Science and Nutrition, The Hebrew University of Jerusalem, Jerusalem, Israel; 2 Smell and Taste Clinic, Department of Otorhinolaryngology, Technical University of Dresden, Dresden, Germany; 3 Institute of Psychology, University of Wroclaw, Wrocław, Poland; 4 Department of Food Science and Human Nutrition, University of Illinois at Urbana Champaign, Urbana, IL USA

**Keywords:** Seven-iTT, Taste strips, Gustatory assessment, Sensory tests, Subjective

## Abstract

**Purpose:**

gustatory ability is a marker of health not routinely tested in the medical practice. The current study wants to assess whether taste strips can be useful to monitor taste function from home.

**Methods:**

we performed simple sensory tests in lab setting vs. unassisted testing at home, and compared the results with self-reports ability to taste and smell. Using paper strips impregnated with sweet, bitter, salty, or sour tastants, and with two trigeminal stimuli (capsaicin, tannins) in high and low concentrations, we assessed gustatory and trigeminal function in 74 participants (47 women) in the lab, where paper strips were administered by an experimenter, and in 77 participants (59 women) at home, where they self-administered the test.

**Results:**

we found that high (but not low) concentration taste strips are correctly identified by vast majority of participants. On average, taste identification, intensity and pleasantness scores did not differ for the 8 taste strips, while identification of capsaicin was significantly better in the lab. Taste identification scores correlated with intensity ratings in both settings (r = 0.56, in the lab, r = 0.48, at home, *p* < 0.005). Self-rated taste ability correlated with self-rated smell ability (r = 0.68, and r = 0.39, p ≤ 0.005), but not with scores in the strips test.

**Conclusion:**

home testing with impregnated taste strips is feasible, and can be used for telemedical purposes.

**Supplementary Information:**

The online version contains supplementary material available at 10.1007/s00405-024-08654-5.

## Introduction

Chemical senses are important markers of health [1–3]. Decline or loss of taste commonly occurs due to aging [4], chemo or radiotherapy treatments, as side-effects of drugs, or after viral infections of the upper respir-atory airways, including infections by SARS-CoV-2 virus which causes coronavirus disease (COVID-19) [5, 6]. Smell and taste functions, assessed by questionnaires or by psychophysical tests were shown to be useful for monitoring the status and recovery of chemosensory impairments, such as those caused by chemotherapy or COVID-19.

Home testing is an unsupervised assessment useful to provide first assistance remotely, as well as in the early detection of chemosensory dysfunction, but it lacks of a detailed physical examination and supervision. Despite these disadvantages, this type of assessments has become a crucial screening procedure for individuals with mobility issues, or those residing in remote areas, and it has been already applied to screen chemosensory function remotely [7, 8].

Despite the importance of gustatory ability as a health marker, it is not evaluated routinely in medical practice. The simplest and fastest way to assess the taste function is to ask the participant directly, as was done extensively during the COVID-19 pandemic [9–11]. However, self-ratings fail to correlate with standardized psychophisical taste tests [12] mostly due to the classical taste-flavor confusion [13]. Among standard tests, regional test and whole mouth procedures [14–16] are relatively time-consuming, ranging from 15 to 40 min per session. On the other hand, identification of tastes using impregnated paper strips (i.e., taste strips) is a common easy and relatively quick method that is used for detecting taste impairment [17, 18]. Our recent development, the “Seven-iTT”, serves as a comprehensive test designed to evaluate both gustatory and somatosensory sensations. Given the correlation between taste function and somatosensation [19, 20], this test comprises four gustatory and two trigeminal stimuli. Such simple screening tools seem to be an option for a self-administered procedure but the reliability of remote procedures remains to be determined.

The main goal of the current study was to assess whether taste strips can be useful to screen taste function from home, and thus be suitable for telemedicine [21]. To this end, the results of this simple standardized test performed in the lab were compared with the results obtained when the taste strips were self-administered at home. At the same time, the relationship between gustatory function measured with the taste strip and subjective self-reported assessment of taste and smell ability was analyzed. An additional aim was to explore the relationship between the sensitivity to oral trigeminal spicy strips and the ratings of their spiciness perception.

## Materials and methods

### Participants

One hundred and twenty-eight participants (Table 1) were recruited on the university campus to perform the test either in an assisted setting in the lab (*n* = 74) or in an unassisted setting at home (*n* = 77). Exclusion criteria consisted of pregnancy, allergies to foods or drugs, hypo/hyperglycemia, kidney failure, and significant heart or blood pressure problems.

**Table 1:** Subjects participating in the study

Cohort	Number of participants	Mean age (SD) in years	Women (% of the group)	Mean (% of the group)	
Lab-tested	74 (49 from group 1, 25 from group 3)	27.4 (5.5)	47 (63.5%)	27 (36.5%)	
Home-tested	77 (54 from group 2, 23 from group 3)	25.1 (3.4)	59 (76.6%)	18 (23.4%)	

These participants were tested in following groups: Group 1—lab condition (*n* = 49, 28.1 ± 5.8 years, 29 females) tested between June and July 2021. Group 2—home condition (*n* = 54, 24.7 ± 2.8 years, 42 females), tested between October and December 2021. To assess the test retest reliability, another group of participants (Group 3) completed both the test in the lab (*n* = 25, 17 females) and at home on the following day (*n* = 23, two participants had technical difficulties completing the test at home), in July 2022. Results that include group 3 are explicitly highlighted in the manuscript. The study was conducted in accordance with the Declaration of Helsinki, and approved by the Ethical Committee for the Use of Human Subjects in Research of the Hebrew University of Jerusalem (Ethical committee file number: 5.21, approved on the 22nd of June 2021). Informed consent was obtained from all subjects involved in the study. Participants in the lab-tested group were compensated for taking part in the experiment.

### Data collection

Data was collected via “Compusense Cloud” (Compusense Inc., Guelph, Ontario, Canada), the lab group answered directly on a desktop computer in a designated room with minimal distraction. The home participants received a kit with the strips and a personal link to the questionnaire. They could perform the test at the time or at any location of their choice. Questions and instructions appeared on the screen in Hebrew for both groups. For the lab group, the experimenter described the instructions before completing the test. While extracting the data for analysis, the identifying username was stripped to provide anonymity of the participants. Timestamps of each of the participant’s responses were recorded for each question to analyze the time of completion of the sensory section.

### Questionnaire

Before completing the sensory test, all participants responded to a questionnaire [22] about general information and medical history (ex. previous surgeries, allergy, etc.). Participants from Group 2 were also asked whether they had contracted COVID-19, as they participated during the height of the COVID-19 pandemic. Additional questions related to self-rated ability to taste and smell were asked using a Likert-type scale ranging from 1 (no ability) to 7 (excellent ability). Twenty-three (Group 3) participants were also asked to rate their ability to sense specific taste modalities on a scale of 1–7. The original questionnaire in Hebrew and its English translation, can be found in Supplementary materials.

### Sensory testing

The participants performed a taste strips identification test including four tastants (bitter, sweet, salty, or sour) and two trigeminally active compounds (capsaicin and tannin) at high and at low concentrations based on the extended version of Seven-iTT [18]. Subsequentially, they rated intensity and pleasantness for each taste strip from 1 to 5.

Sample presentation followed the same order as in [18], which allows for further comparison between the two experiments, with gustatory stimuli preceding trigeminal exposure [23, 24], and low concentrations preceded the high concentrations [17]. The concentrations of the solutions that were used to impregnate the paper strips are listed in Table 2. Only group 1 tasted tannin-impregnated papers, which were later excluded from analyses and from further tests due to its brown color which was visually distinct from all other strips. Strips were prepared in the Technische Universität Dresden.

**Table 2:** Taste strips impregnated with compounds and concentrations

Strip impregnation		Gustatory				Trigeminal	
		Sour	Sweet	Bitter	Salty	Spicy	Astringent
Compound		Citric acid (g/ml)	Sucrose (g/ml)	Quinine hydrochloride (g/ml)	Sodium chloride (g/ml)	Capsaicin (g/ml)	Tannin (g/ml)
Concentration	Low	0.05	0.05	0.0004	0.016	$2.47\times {10}^{-5}$	0.1
	High	0.30	0.40	0.0060	0.250	$2.47\times {10}^{-4}$	0.2

For the lab group, the experimenter placed the strip at the center of the anterior two-thirds of the tongue, a highly innervated, easily accessible, and frequently affected by gustatory impairments part of the tongue [25]. The participants were asked to keep the strip in their mouth for a few seconds or until they felt a taste or sensation, then they were allowed to freely move their tongue and answered whether they sensed a taste. If so, subjects were asked to identify the taste, and rate its intensity and pleasantness. The tested strips were visually identical and labeled with random 3-digit codes. The home participants have received kits that included the strips, and a link to the online program to complete the test. They self-administered the strips with their eyes open, following the same ordered as in the lab test.

For each correctly identified strip (high and low concentration) participant received one point, resulting in a maximum score of 8 points for the basic tastes, and of 2 points for capsaicin, which was calculated separately. Rating scores ranged from very unpleasant or no intensity (both scored with 0), to very pleasant or very intense (score of 5). Average intensity and average pleasantness were calculated for the four basic taste modalities (i.e., without capsaicin). For average intensity, if the participant did not feel any taste for a strip, 0 intensity was used as the intensity score. Strips for which a taste was sensed (either correctly identified or not), were included in the calculation of the average intensity and average pleasantness scores. For example, if the participant has felt a taste for three of the eight strips, then the average score for that person will be the sum of the intensity of the three strips, divided by eight; while the average pleasantness score will be the sum of pleasantness for the three strips, divided by three.

### Statistical analysis

The testing power of the experiment was determined using G power 3.1.9.7 software [26]. We ran power analysis based on a reference experiment of assisted and unassisted taste strips comparison [27], and estimated the needed sample size as 134, using Wilcoxon-Mann-Whittney-test for two groups with an alpha of 0.05, power of 0.8. Data were analyzed using python, version 3.6.9 in Google Colab Environment. For statistical tests, the SciPy package was used with version 1.8.0. Values are presented as the mean ± Standard Deviation (SD). Both lab and home group results were tested for departure from normality. The home test scores for strip identification score, intensity score, and pleasantness score were not normally distributed and were analyzed using the Mann–Whitney U test. Pearson’s correlation coefficient (r) was used to calculate correlations. To compare correlations, a conversion from correlation to z score was done using Fisher r-to-z transformation depicting the z, and two-tailed p-value was used. The threshold for statistical significance was set to *p* < 0.05. Bonferroni correction was applied when conducting multiple comparisons of 8 taste strips, hence corrected p-value was set to 0.00625. Significance annotations along the paper are * for *p* < 0.05, ** for *p* < 0.01, *** for *p* < 0.001, and **** *p* < 0.0001.

## Results

**Fig. 1:**
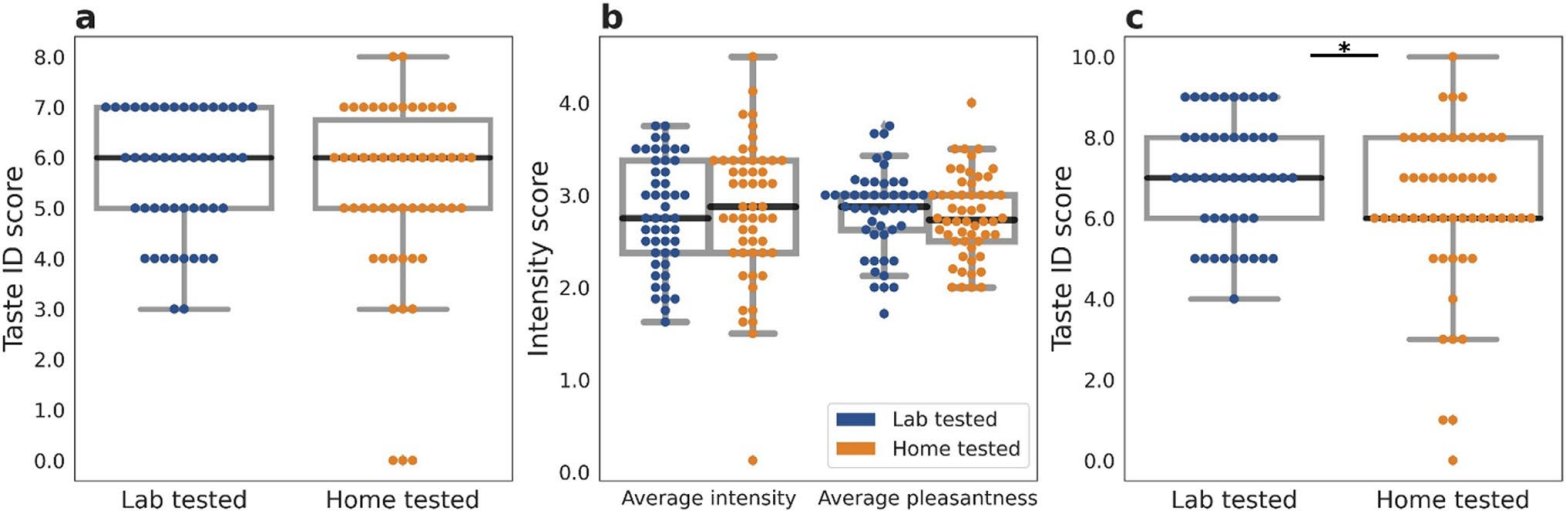
Taste ID and characteristics among the lab (group 1) and home (group 2) group participants. **a**) Taste ID score distribution and **b**) average intensity and pleasantness ratings over the 8 strips. **c**) Taste ID score distribution including capsaicin strips. Comparing lab and home groups colored in blue and orange respectively, the black line indicates the median, while the gray box indicates the quartiles of the dataset. * means *p*-value < 0.05

Taste identification (ID), pleasantness, and intensity scores were compared between the lab and home groups (groups 1 (lab) and 2 (home)). Identification score distributions between the two cohorts were similar (*p* > 0.05; Mann–Whitney U test). Intensity and pleasantness distributions were also similar for home and lab tests (*p* > 0.05) (Fig. 1a,b). Interestingly, including in the analysis the low and high concentration capsaicin strips showed that the results in the lab and at home were similar for intensity and pleasantness, but taste identification was higher for the lab 7.06 ± 1.45 compared to home 6.24 ± 2.02 (*p* = 0.048) (Fig. 1c).

Taste score and intensity were similar for both genders, but in the lab group males found the strips slightly more pleasant tasting (average pleasantness of 3.00 ± 0.35) than females did (average pleasantness rating of 2.71 ± 0.48) (*p* = 0.012).

**Fig. 2:**
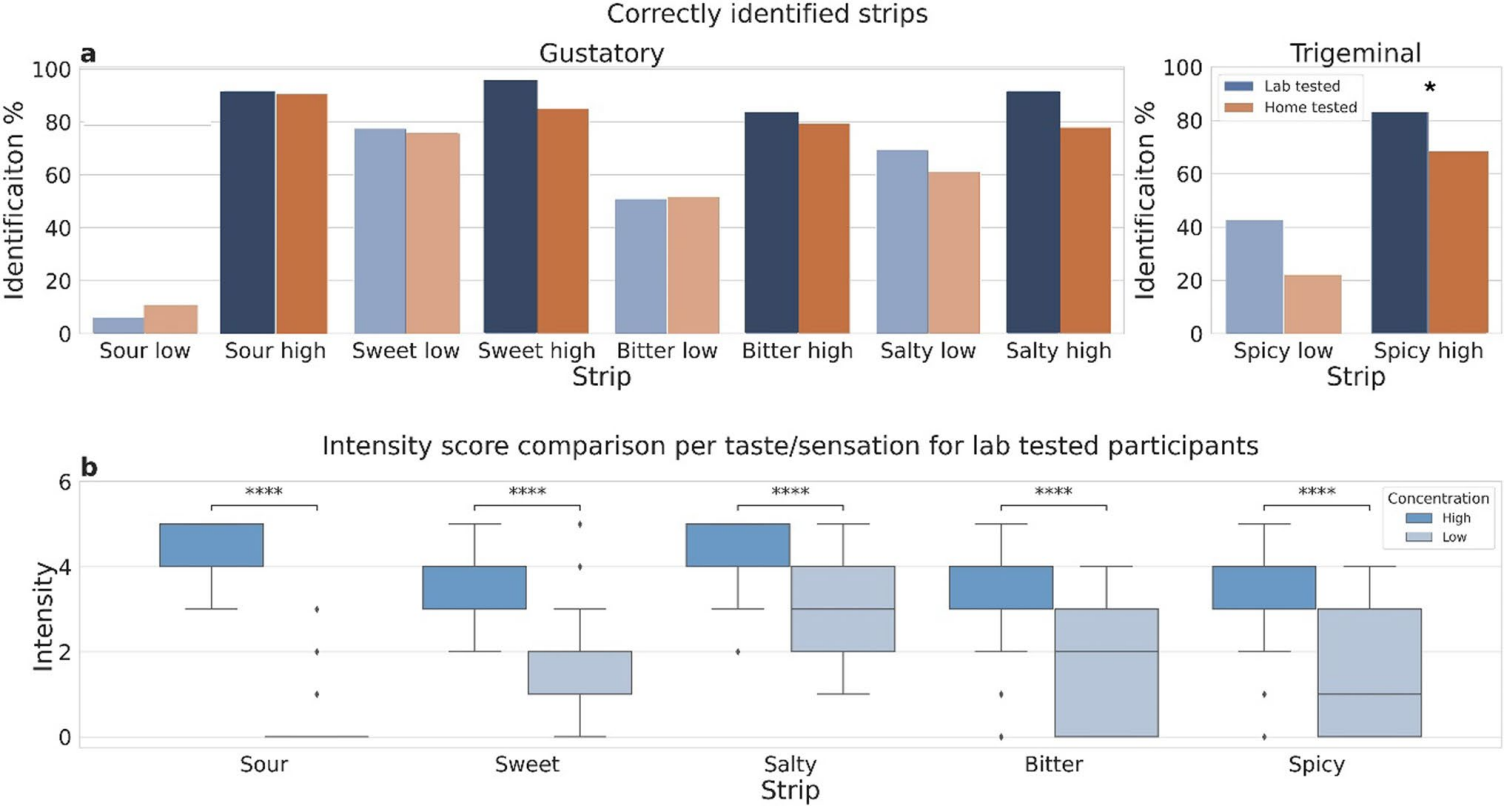
**a** Percent of correctly identified strips, blue for lab (Group 1) and orange for home (Group 2) testing, * means *p*-value = 0.00625. High-concentration strips are colored in darker shades, compared to lower concentrations. **b** Intensity of each strip comparing high against low concentrations for participants tested in the lab. **** means *p*-value < 0.001.

The low-concentrated sour taste strip was correctly identified only by a few participants in both groups (Fig. 2A). The sweet strip was the best identifiable strip among the low-concentration strips. The same results were found for group 3 (correct identification in lab conditions: 82.6%; at home: 69.6%; *p* = 0.31). The biggest difference in identification ability was found for the low concentration spicy strips, with 42.6% for lab tested group, and 22.2% for home tested group (*p* = 0.0085). The other low-concentrated strips were similarly identified by both groups (bitter, 51.0% lab, 51.9% home, *p* = 0.94; salty, 69.4% lab, 61.1% home, *p* = 0.38).

Consistent with the trend found for low concentration capsaicin strip, the correct identification of the high concentration capsaicin strip was significantly lower at home (68.5%) than in the lab (83.3%, *p* = 0.0036).

As expected, intensity scores of high-concentrated strips were significantly higher than the low concentration strips for the same taste modality (sour: *p* = 1.05 × 10^–26^; sweet: *p* = 4.71 × 10^–17^; bitter: *p* = 3.33 × 10^–10^; salty: *p* = 1.03 × 10^–11^; spicy: *p* = 1.25 × 10^–10^) (Fig. 2B for the lab group).

### Correlation with subjective self-reporting

**Fig. 3:**
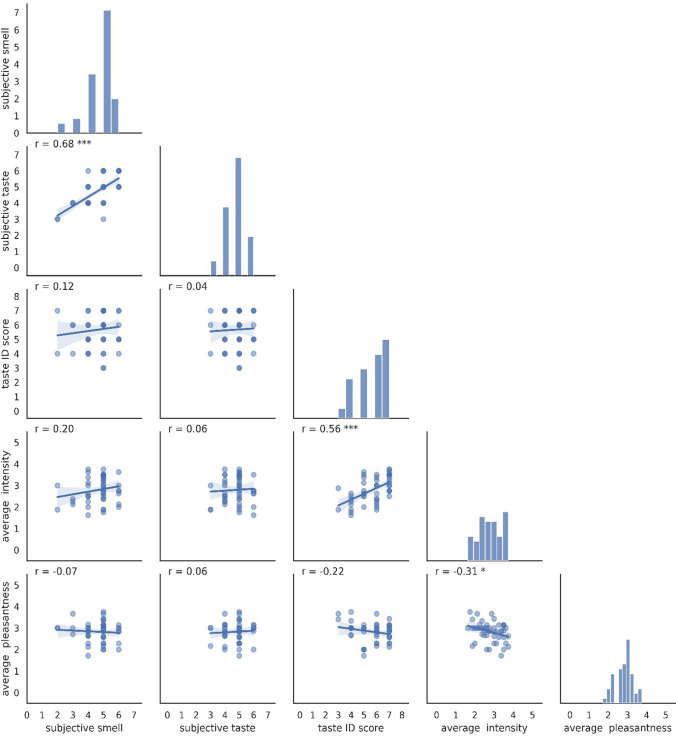
Correlations between the self-rated abilities to smell and taste (subjective smell and taste), taste identification score (taste ID score), and averaged across the four basic taste modalities regarding intensity and pleasantness ratings individually (average pleasantness and intensity), represented by scatter plots including Pearson’s correlation coefficient (r) at the top left of each panel, and regression line. Histograms of the diagonal panels of the figure present the frequency of scores, with the x-axis indicating the score and the y indicating the count of participants. Shown for lab group (group 1, *n* = 49), * means p ≤ 0.05, *** means p ≤ 0.001

Self-reported taste ability was strongly correlated with self-reported smell ability for the lab group (Fig. 3) and exhibited a weaker correlation with for the home group (Suppl. Figure 1) (r = 0.68, *p* = 8.30 × 10^–8^ and r = 0.38, *p* = 0.004, respectively). These coefficients of correlations were significantly different (z = 2.11, *p* = 0.017). In contrast, subjective taste ability had weak or negligible correlations with taste ID, intensity ratings, or pleasantness ratings, for both the lab and the home group. Taste ID score was correlated with average intensity, especially in the lab (lab: r = 0.56, *p* = 3.27 × 10^–5^; home: r = 0.48, *p* = 2.35 × 10^–4^), while it was not correlated with average pleasantness. A slightly negative correlation between average intensity and average pleasantness was shown for the lab group (lab: r = -0.31, *p* = 0.028; home: r = -0.11, p = 0.44). Adding capsaicin results, taste ID and intensity increased the correlation coefficient to 0.7, while pleasantness to intensity correlation became -0.23, with the rest of the correlations remaining practically unchanged.

### Test re-test consistency and correlations

The similarity between the average home and the lab results are striking, given the difference in the setting, the way of administration, and the different participants at home and in the lab. We therefore carried out a test–retest consistency assessment for an additional group of participants, group 3, that completed both the lab and then the home test.

**Fig. 4:**
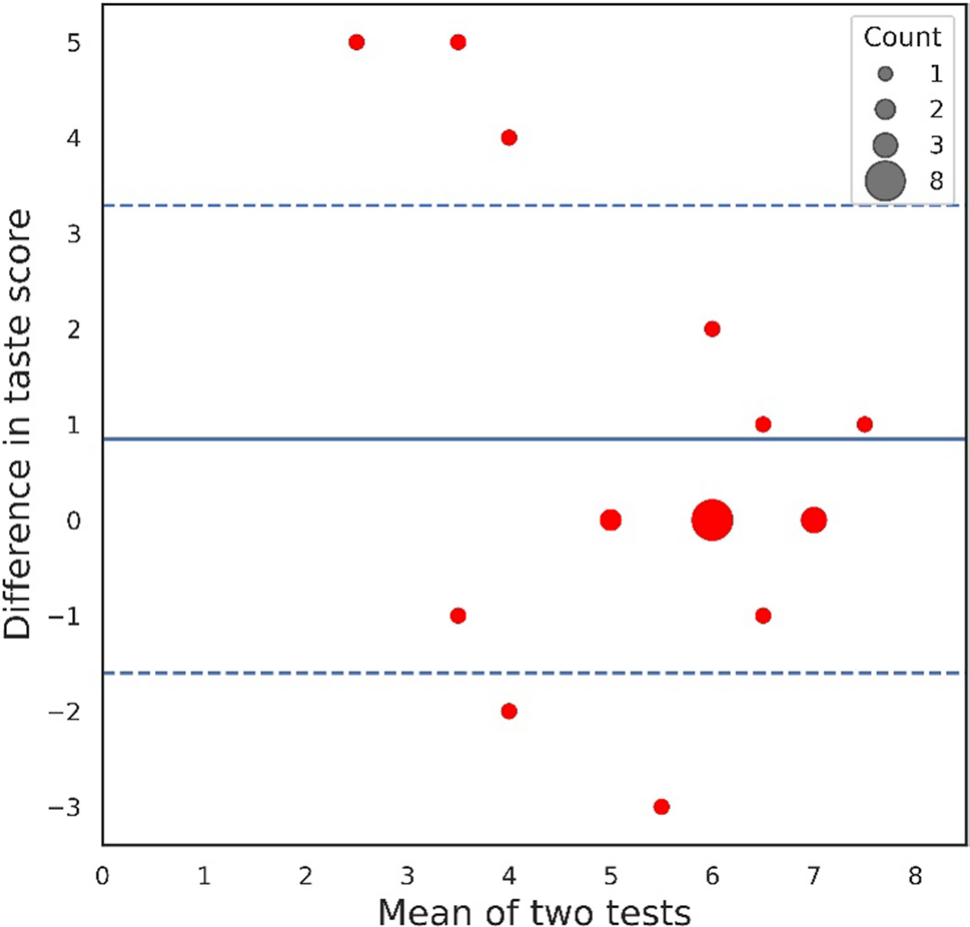
Bland–Altman plot. Test–retest consistency for taste identifications scores of the participants that have done the test both in the lab and at home (*n* = 23), on the Y axis is the difference between the score in the first test subtracted by the second, while the X axis indicates the mean of taste score between the two test repetitions. The mean of the difference between the two tests' taste scores is colored in blue, with one standard deviation from the mean shown in a dashed line. Count of participants with identical scores is shown by the size of the point

The mean of the first scores deducted by the repetition scores was 0.85 ± 2.44, five participants scored outside of one standard deviation interval from the mean, in three cases they performed better at the lab and two had better scores at home as shown in Fig. 4. Their scores for ID (lab: 6.56 ± 1.53; home: 6.65 ± 1.72), average pleasantness (lab: 2.53 ± 0.63; home 2.6 ± 0.56), and average intensity (lab: 2.91 ± 0.40; home: 2.99 ± 0.40) were similar between the two repetitions (*p* ≥ 0.51). Like the other groups, group 3 participants identified low-concentration taste strips similarly in the lab and at home (bitter, 43.5% lab, 39.1% home, *p* = 0.78, salty, 70.0% lab, 82.6% home *p* = 0.31).

**Fig. 5:**
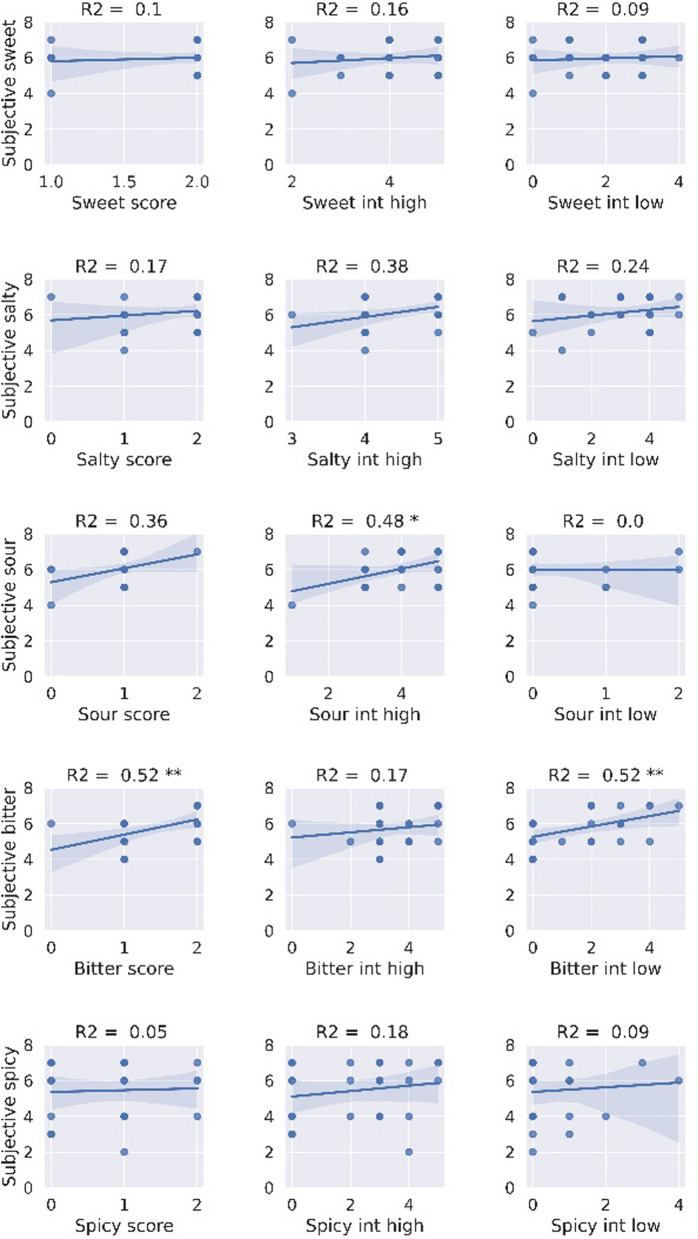
Correlations of participants in group 3 (*n* = 25) regarding lab group scores, between each taste modality self-rating and taste strip scores, at low and high intensity. int = intensity; * means p ≤ 0.05, ** means p ≤ 0.01

Participants in group 3 were also asked to rank subjective ability for individual taste modalities. The highest correlation was found for the self-reported ability to sense bitter taste with the strip identification score calculated for low and high bitter concentrations (r = 0.52, *p* = 0.007), and with intensity of the lower concentration of the bitter strip (r = 0.52, *p* = 0.008) (Fig. 5). Another correlation found was between the subjective assessment and the taste intensity score of the sour high concentration strip (r = 0.48, *p* = 0.014).

We have also compared the main metrics of taste ID, average intensity, and average pleasantness. The results of those participants were similar to the scores of groups 1 and 2. Identification scores were 5.76 ± 1.23 when completing the test in the lab, and 5.65 ± 1.56 at home (*p* = 0.92). Average pleasantness was also very similar, 2.81 ± 0.68 at the lab, and 2.79 ± 0.56 at home (*p* = 0.66), and average intensity followed a similar pattern, 2.98 ± 0.39 at lab conditions, and 3.00 ± 0.46 at home. Notably, adding group 3 participants to groups 1 and 2, the results reported for these groups did not change. For example, the correlations for lab group subjective taste and subjective smell correlation remain the highest (r = 0.73, *p* = 1.02 × 10^–13^), and average intensity and taste identification score correlation is also high (r = 0.63, *p* = 2.09 × 10^–9^).

### Time to complete sensory test

For assessing the practicality of the tests, we checked the time to completion for the 8 taste strips. We found that the time distribution did not significantly differ between the home and the lab setting (including all three groups) (Suppl. Figure 2). Home tested average time to complete the sensory taste test was 8 min and 40 s (SD 3 min and 28 s), while the time required for the lab test was 8 min and 45 s (SD 2 min and 5 s) (*p* = 0.2). The duration required to evaluate an individual taste strip, including time for palate cleansing with water prior to proceeding to the subsequent strip, can be estimated as one minute per strip on average and is the same at home and in the lab.

## Discussion

The present study explored the taste identification ability, intensity, and pleasantness of the basic taste modalities (sour, sweet, salty, and bitter) in young Israeli participants and similar results were obtained in the lab and at home. On the contrary, paper strips impregnated with capsaicin solutions were differently identified in the two settings. Subjective self-assessment of taste ability mostly did not correlate with psychophysical results.

Our study revealed no significant differences in taste ID, average intensity, and average pleasantness between groups 1 and 2. Adding group 3 did not change the result. This means that a simple taste strips test holds promising potential as a self-administered test at home. This can be of great value when looking ahead to monitoring taste status using telemedicine. Indeed, as the COVID-19 pandemic brought the research of smell and taste to the forefront, new quick tests of the sense of smell have been introduced, for example, SCENTinel and ArOMa-T [28–30], while quick taste testing still remains less common. Low concentrations of sour and spicy strips were poorly identified by the participants in this study, with particularly low success for low concentrations of spicy strips for the home group. This suggests that low concentrations of sour, spicy, and perhaps bitter strips are not suitable for fast monitoring of gustatory function in the clinic, since they are poorly identified even by young healthy population. Interestingly, higher percentage (22.7%) of the 154 German healthy participants tested in Mastinu et al. study [18] were able to identify the same low-concentration sour strips, compared to the 8.7% of the Israelis. Thus, low-concentration strips may provide an interesting comparative tool from cross-cultural studies and warrant further study.

The trigeminal stimuli were explored in a preliminary manner, because only capsaicin could be used as a stimulus, while tannin strips were excluded due to their distinctive color. The identification of capsaicin strips at both concentrations was less successful at home, as manifested not only in significant differences between lab and home for each capsaicin strip, but also in in the composite identification that includes capsaicin. This is intriguing and requires a follow-up study to explore the underlying reasons, possibly related to compliance, for the observed difference between capsaicin perception in the lab and the home settings.

The lack of correlation between self-reported ability and the actual scores on taste ID were rather striking. While the ID and mean intensity were correlated, as expected, the overall subjective self-reported taste function had a low or negligible correlation with the measured outcomes (identification, intensity, and pleasantness) of taste strip tasting. This further supports the notion that self-reported taste/flavor is affected by sensations other than the taste of basic taste modalities, and that information from psychophysical tests and from subjective reporting is not redundant, but may rather be complimentary.

Subjective reports on individual taste modalities tested in group 3 also had a very low correlation with objective measures for that taste modality, except for bitter low concentration and sour high concentration. A possible explanation for some correlation with the bitter strip scores is the wider variation in bitterness perception, while in other taste modalities, most of the healthy participants had similar (high) scores. The correlation between the high concentration sour strip and the self-reported sour sensitivity was also significant. It will be interesting to further study subjective assessment of sensitivity towards individual taste modalities and psychophysical ratings in larger cohorts, using a larger range of concentrations and utilizing more sensitive ranking scales.

Interestingly, the analysis of different scores for the same individual demonstrated the highest correlation between self-reported smell and self-reported taste. Since in Hebrew the same word is used for “taste” and “flavor”, the confusion may be due to the dominant role olfaction plays in the perception of flavors [31]. It will be interesting to expand such types of correlations in the future, by adding additional trigeminal, as well as olfactory stimuli. Perhaps, a combination of the different stimuli identification and intensity may provide a better fit to the subjective self-assessment of chemosensory abilities.

Despite useful insights obtained in this study, several limitations should be kept in mind. Rather crude scales were used: the self-rating scales were Likert 1–7 scales and the objective identification scores were 0–2. The presentation order of the strips was not randomized, and the sample size was not particularly large. Nevertheless, the current study provides multiple interesting insights about the testing at home and in the lab, and sets a useful initial baseline reference in a young healthy population. This can lay the foundation for further studies focused on standardizing a simple tool that can track gustatory ability along with olfactory ability in at-home settings [32]. Such a tool could become helpful in monitoring sensory impairments associated with different conditions, including diabetes mellitus, head trauma, and aging [33, 34].

## Conclusions

Taste ID for high and low concentrations of sweet, sour, salty, bitter and capsaicin in the impregnated strips test was consistent for all taste quality in the lab and the home setting, but spicy was identified better in the lab condition. The results are consistent across the two settings also for average intensity and pleasantness, while they were not correlated with self-ratings. The test appears to be a feasible method for remote taste testing, with high test–retest reliability, and can be applied for preliminary distant screening.

## Supplementary Information

Below is the link to the electronic supplementary material.

Supplementary file1 (DOCX 37 KB)

Supplementary file2 (DOCX 282 KB)

## Data Availability

The datasets generated during and/or analyzed during the current study are available from the corresponding author upon request.
