# Supplementary material for: Home screening of taste and oral trigeminal function: a feasibility study
Source: Eur Arch Otorhinolaryngol. 2024 Apr 17;281(9):4835–44. doi: 10.1007/s00405-024-08654-5 (PMC11392964; doi:10.1007/s00405-024-08654-5)
Supplement: Supplementary file 1 — Supplementary file1 (DOCX 37 KB) [file 405_2024_8654_MOESM1_ESM.docx]

The participants have completed the questionnaire in hebrew, translated from the enlgish questionnaire below.

**Questionnaire**

Medical History

Date: ................................ Patient Nummer: investigator:

Please tick the appropriate box. **General information**

height(cm) .......... weight (kg) .................. Age ................. (J) □ female □ male

profession**:** Education:

currently employed as: .......................................................................................................... ...................

**Medical history**

Exist or existed one of the following **diseases**?

□ no □ yes, the following

□ head trauma ? □ frequent cold / influenza ?

□ frequent sinusitis ? □ nasal polyposis ?

□ allergic rhinitis ? □ problems breathing through the nose ?

□ headache ? □ runny nose ?

□ post-nasal drip ? □ snoring ?

□ disorder of nervous system / cerebral disease ? □ hepatitis ?

□ diabetes mellitus ? □ kidney disease?

□ thyroid **hyper**function ? □ thyroid **hypo**function ?

□ and other (which one?) ..............................................................................................................................................

..............................................................................................................................................................................

Have you already had an operation on your head? □ no

yes □ paranasal sinuses - when ? □ nasal septum- when ?

□ nasal turbinates - when ? □ palatal tonsil - when ?

□ pharyngeal tonsil (“polyps”) - when ? □ middle ear- when ?

□ dental surgery (e.g. wisdom tooth extraction) -when ? ...........................

□ other operations -when ? ............................... ............

Do you drink **alcohol** □ no □ sometimes □ regularly

Do you **smoke**? □ no, never before

□ no, not for ............. years □ yes since .............years

How is your **salivation**? □ too much □ normal

□ too little – I have a dry mouth □ much too little – I have a very dry mouth

Are you able to taste sweet, sour, salty and bitter? □ yes □ no

If you put something in your mouth, does it always produce the same taste (bitter, salty, or metallic), regardless what it is? □ yes □ no

Do you **permanently** taste one of the following sensations in your mouth: bitter, sour, salty or metallic? □ yes □ no

Self-assessment

How do you rate your **ability to taste**?

excellent-------------------------------------- □

very good --------------------------------------------- □
good --------------------------------------------- □
normal ---------------------------------------------- □
bad --------------------------------------- □
very bad -------------------------------------- □
no perception ------------------- □

How do you rate your **ability to smell**?

excellent-------------------------------------- □

very good --------------------------------------------- □
good --------------------------------------------- □
normal ---------------------------------------------- □
bad --------------------------------------- □
very bad -------------------------------------- □
no perception ------------------- □

How do you rate the perception of **spiciness**?

excellent-------------------------------------- □

very good --------------------------------------------- □
good --------------------------------------------- □
normal ---------------------------------------------- □
bad --------------------------------------- □
very bad -------------------------------------- □
no perception ------------------- □

How do you rate the perception of **astringency**?

excellent-------------------------------------- □

very good --------------------------------------------- □
good --------------------------------------------- □
normal ---------------------------------------------- □
bad --------------------------------------- □
very bad -------------------------------------- □
no perception ------------------- □

How satisfied are you with your ability of taste?

not very

satisfied satisfied

Please rate the strip __________

I perceived nothing □ / perceived something □

The **intensity** is The **sensation** is It tastes

very low □ very unpleasant □ sweet □

low □ unpleasant □ sour □

medium □ neutral □ salty □

strong □ pleasant □ bitter □

very strong □ very pleasant □ spicy □

„numb“/astringent □

Please rate the strip __________

I perceived nothing □ / perceived something □

The **intensity** is The **sensation** is It tastes

very low □ very unpleasant □ sweet □

low □ unpleasant □ sour □

medium □ neutral □ salty □

strong □ pleasant □ bitter □

very strong □ very pleasant □ spicy □

„numb“/astringent □

Please rate the strip __________

I perceived nothing □ / perceived something □

The **intensity** is The **sensation** is It tastes

very low □ very unpleasant □ sweet □

low □ unpleasant □ sour □

medium □ neutral □ salty □

strong □ pleasant □ bitter □

very strong □ very pleasant □ spicy □

„numb“/astringent □

Please rate the strip __________

I perceived nothing □ / perceived something □

The **intensity** is The **sensation** is It tastes

very low □ very unpleasant □ sweet □

low □ unpleasant □ sour □

medium □ neutral □ salty □

strong □ pleasant □ bitter □

very strong □ very pleasant □ spicy □

„numb“/astringent □

Please rate the strip __________

I perceived nothing □ / perceived something □

The **intensity** is The **sensation** is It tastes

very low □ very unpleasant □ sweet □

low □ unpleasant □ sour □

medium □ neutral □ salty □

strong □ pleasant □ bitter □

very strong □ very pleasant □ spicy □

„numb“/astringent □

Please rate the strip __________

I perceived nothing □ / perceived something □

The **intensity** is The **sensation** is It tastes

very low □ very unpleasant □ sweet □

low □ unpleasant □ sour □

medium □ neutral □ salty □

strong □ pleasant □ bitter □

very strong □ very pleasant □ spicy □

„numb“/astringent □

Please rate the strip __________

I perceived nothing □ / perceived something □

The **intensity** is The **sensation** is It tastes

very low □ very unpleasant □ sweet □

low □ unpleasant □ sour □

medium □ neutral □ salty □

strong □ pleasant □ bitter □

very strong □ very pleasant □ spicy □

„numb“/astringent □

Please rate the strip __________

I perceived nothing □ / perceived something □

The **intensity** is The **sensation** is It tastes

very low □ very unpleasant □ sweet □

low □ unpleasant □ sour □

medium □ neutral □ salty □

strong □ pleasant □ bitter □

very strong □ very pleasant □ spicy □

„numb“/astringent □

Please rate the strip __________

I perceived nothing □ / perceived something □

The **intensity** is The **sensation** is It tastes

very low □ very unpleasant □ sweet □

low □ unpleasant □ sour □

medium □ neutral □ salty □

strong □ pleasant □ bitter □

very strong □ very pleasant □ spicy □

„numb“/astringent □

Please rate the strip __________

I perceived nothing □ / perceived something □

The **intensity** is The **sensation** is It tastes

very low □ very unpleasant □ sweet □

low □ unpleasant □ sour □

medium □ neutral □ salty □

strong □ pleasant □ bitter □

very strong □ very pleasant □ spicy □

„numb“/astringent □

Please rate the strip __________

I perceived nothing □ / perceived something □

The **intensity** is The **sensation** is It tastes

very low □ very unpleasant □ sweet □

low □ unpleasant □ sour □

medium □ neutral □ salty □

strong □ pleasant □ bitter □

very strong □ very pleasant □ spicy □

„numb“/astringent □

Please rate the strip __________

I perceived nothing □ / perceived something □

The **intensity** is The **sensation** is It tastes

very low □ very unpleasant □ sweet □

low □ unpleasant □ sour □

medium □ neutral □ salty □

strong □ pleasant □ bitter □

very strong □ very pleasant □ spicy □

„numb“/astringent □

Hebrew Questionnaire

אנא ענה/עני על השאלות הבאות:

1. גובה (בסנטימטר, לדוגמא 174):
2. משקל (בקילוגרם):
3. גיל:
4. אנא ציין/צייני את המין שלך:

זכר

נקבה

מעדיף/מעדיפה לא לציין

אחר

1. רקע לימודי:
2. רקע לימודי:
3. עבודה נוכחית:
4. היסטוריה רפואית

דלקת סינוסים כרונית

זעזוע ראש

נזלת מרובה

כאב ראש

סכרת

בעיה עצבית

פעילות נמוכה של בלוטת התריס

פעילות יתר של בלוטת התריס

פוליפ

שפעת כרונית

צינון

קשיי נשימה מהאף והפה

צהבת

נחירה

בעיה בכליות

אחר (פרט.י)

אף אחד מהאפשרויות המוצגות

מעדיף/מעדיפה לא לציין

1. האם עברת ניתוח באזור הראש?

ניתוח בחללי האף – אם כן מתי

לא

ניתוח אנדואיד (שקד האף) - אם כן מתי

ניתוח קונכיות האף - אם כן מתי

ניתוח במחיצת האף - אם כן מתי

ניתוח דנטלי (לדוגמא עקירת שן בירה) - אם כן מתי

ניתוח באוזן התיכונה - אם כן מתי

ניתוח בשקדי החך - אם כן מתי

מעדיף/מעדיפה לא לציין

1. האם את/ה שותה אלכוהול?

לא

לפעמים

לעיתים קרובות

מעדיף/מעדיפה לא לציין

1. האם את/ה מעשן/ת?

לא, מעולם לא עישנתי

לא, כבר לא מעשן למשך: (שנים)

כן, מעשן למשך: (שנים)

1. איך היית מגדיר/ה את רמת ייצור הרוק שלך?

גבוהה מדי

רגילה

נמוכה – יש לי פה יבש

מאוד נמוכה – יש לי פה יבש מאוד

1. האם את/ה מסוגל/ת לחוש את הטעמים מתוק, חמוץ, מלוח, ומר?

כן

לא

1. כאשר את/ה טועם/ת משהו, האם את/ה תמיד חש/ה **בטעם זהה** (מר, מלוח או מתכתי), לא משנה מה את/ה טועם/ת?

כן

לא

1. האם את/ה חש/ה **באופן תמידי** את אחד הטעמים הבאים בפיך : מר, חמוץ , מלוח או מתכתי?

כן

לא

1. איך היית מדרג/ת את **היכולת שלך לטעום**?

מעולה

טובה מאוד

טובה

בינונית

גרועה

גרועה מאוד

אין יכולת

1. איך היית מדרג/ת את היכולת שלך להריח?

מעולה

טובה מאוד

טובה

בינונית

גרועה

גרועה מאוד

אין יכולת

1. איך היית מדרג/ת את **הרגישות שלך לתחושת חריפות**?

מעולה

טובה מאוד

טובה

בינונית

גרועה

גרועה מאוד

אין יכולת

1. איך היית מדרג/ת את **הרגישות שלך לתחושת עפיצות**(בדומה תחושה שלאחר שתיית יין יבש)?

מעולה – רגישות גבוהה מאוד

טובה מאוד

טובה

בינונית

גרועה

גרועה מאוד – רגישות נמוכה מאוד

אין יכולת לחוש עפיצות

1. כמה את/ה מרוצה מהיכולת שלך לטעום?


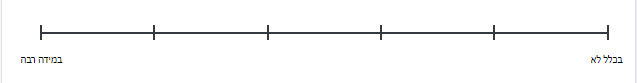


קטע הטעימה:

אנא קרא/י את ההנחיות לפני לביצוע הטעימה:

על הטועם/טועמת לעצום את עיניו/עיניה

 להכניס את הצד העבה של מקל הנייר על מרכז הלשון ולסגור את הפה

 למצוץ את מקל הנייר למשך מספר שניות על מנת לחוש בטעם.

במידה וטעמת טעם, יהיה עלייך לענות על מספר שאלות:

טעם/י את דוגמא מספר :

1. האם חשת בטעם:

חשתי בטעם

לא חשבתי בטעם

1. דרג/דרגי את חוזק הטעם:

חזק מאוד

חזק

בינונית

נמוכה

נמוכה מאוד

1. דרג/דרגי את התחושה בעקבות טעימת הנייר:

מאוד נעימה

נעימה

ניטרלית

לא נעימה

מאוד לא נעימה

1. ציין/צייני מהו הטעם/תחושה שחשת:

מתוק

מר

מלוח

חמוץ

חריף

עפיץ (בדומה לתחושה שלאחר שתיית יין יבש)

נא לשטוף את הפה עם מים לקראת הדוגמא הבאה
