# Supplementary material for: Home screening of taste and oral trigeminal function: a feasibility study
Source: Eur Arch Otorhinolaryngol. 2024 Apr 17;281(9):4835–44. doi: 10.1007/s00405-024-08654-5 (PMC11392964; doi:10.1007/s00405-024-08654-5)
Supplement: Supplementary file 2 — Supplementary file2 (DOCX 282 KB) [file 405_2024_8654_MOESM2_ESM.docx]

Correlations between self-rated scores and taste test scores

**
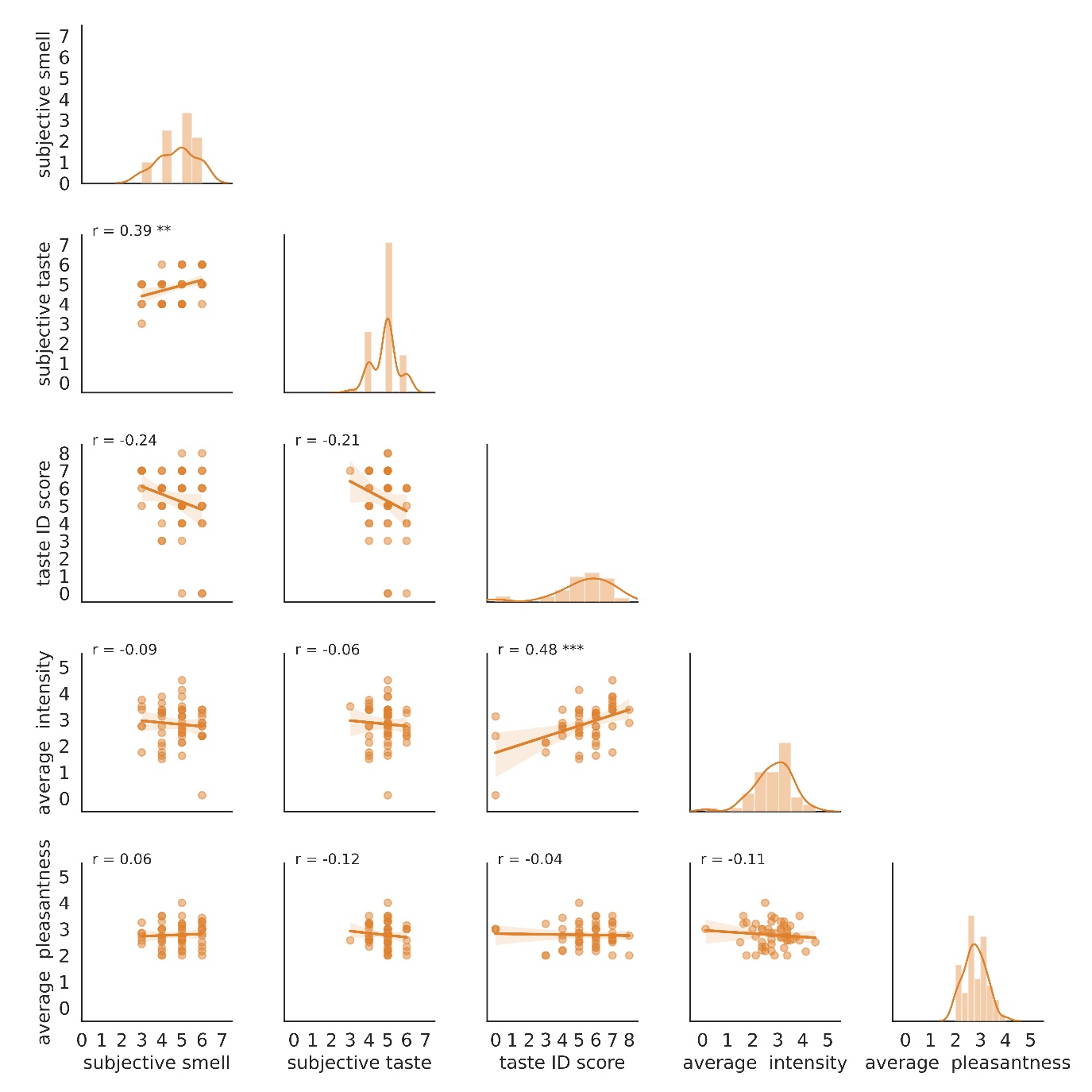
**

**Supplementary Fig. 1** Correlations between the self-rated abilities to smell and taste (subjective smell and taste), taste identification score (taste ID score), and average across all of the strips regarding intensity and pleasantness ratings individually (average pleasantness and intensity), represented by scatter plots including Pearson’s correlation coefficient (r) at the top left of each panel, and regression line. Histograms the diagonal panels of the figure present the frequency of scores, with the x axis indicating the score and the y indicating the count of participants. Shown for the home group (group 2, *n* = 54). ** means *p* ≤ 0.01 and *** means *p* ≤ 0.001

**
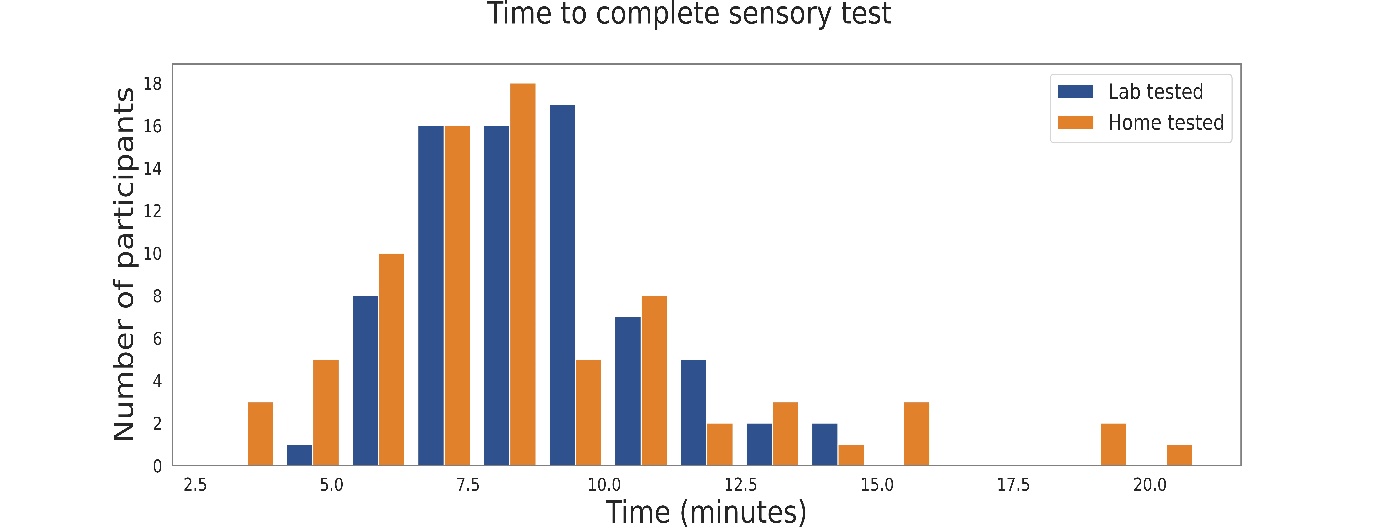
**

**Supplementary Fig. 2** Distribution of time to complete the sensory test for all three groups, lab test in blue, home test colored in orange.
